# Corrosion behavior of multi-layer friction surfaced structure from dissimilar aluminum alloys

**DOI:** 10.1038/s41598-024-60431-w

**Published:** 2024-04-30

**Authors:** Eduardo Antunes Duda, Zina Kallien, Sabrina da Silva Soares, Tárique Hernandez Schneider, Henrique Ribeiro Piaggio Cardoso, Guilherme Vieira Braga Lemos, Tiago Falcade, Afonso Reguly, Benjamin Klusemann

**Affiliations:** 1 Solid State Materials Processing, Institute of Material and Process Design, Helmholtz-Zentrum Hereon, Max-Planck-Straße 1, 21502 Geesthacht, Germany; 2 Laboratório de Metalurgia Física (LAMEF), Universidade Federal do Rio Grande do Sul (UFRGS), Porto Alegre, RS Brazil; 3 Programa de Pós-Graduação em Engenharia de Minas, Metalúrgica e Materiais, PPGE3M, Universidade Federal do Rio Grande do Sul (UFRGS), Porto Alegre, RS Brazil; 4 Universidade Federal de Santa Maria, UFSM, Cachoeira do Sul, RS Brazil; 5 Institute for Production Technology and Systems, Leuphana University Lüneburg, Universitätsallee 1, 21335 Lüneburg, Germany

**Keywords:** Friction surfacing, Corrosion, Solid state layer deposition, Aerospace engineering, Characterization and analytical techniques, Microscopy

## Abstract

Friction surfacing (FS) is a solid-state coating technology for metallic materials, where the deposition of a consumable material on a substrate is enabled via friction and plastic deformation. The deposited layer material commonly presents a significantly refined microstructure, where corrosion could be an issue due to this grain refinement within the layer deposited, possibly creating micro galvanic pairs. The present work investigates the corrosion behavior of the FS deposited material as well as stud base material and substrate using cyclic polarization tests and open circuit potential (OCP) monitoring. Comparing the FS deposited material and the respective consumable stud base material (both AA5083), the grain size is correlated with the results from the corrosion tests, where the deposited material shows more equiaxed and refined grains in comparison to the stud base material. The cyclic potentiostatic polarization tests showed that the stud base material is more resistant to pitting nucleation presenting smaller pits and a lower amount of pits compared to deposited material and substrate. As a complement to OCP test, the stud base material is also more stable on a chloride solution compared to the substrate and the deposited material.

## Introduction

Friction surfacing (FS) is a technique for solid state layer deposition of a metallic consumable stud material onto a substrate below the materials’ melting temperature^1^. For the deposition via FS, the consumable stud material is positioned above the substrate and it experiences a defined rotational speed and axial force. The stud is pressed onto the substrate and due to frictional heat at the materials’ interface, the stud’s tip starts to deform and plasticize^2^. A relative translational movement between the plasticized stud and the substrate material at a defined travel speed enables the deposition of a layer of the consumable stud material onto the substrate. Using the FS technique, single layers can be used to locally add material to a structure. Additionally, FS also shows potential for repair applications, which was for instance presented by Damodaram et al.^3^, who showed the successful filling of cracks via FS layer deposition. The process is not limited to single layers as process variants of multi-track friction surfacing (MTFS) and multi-layer friction surfacing (MLFS) allow the deposition of multiple layers adjacent to and on top of each other, respectively. Therefore, the FS deposition technique shows strong potential for solid state additive manufacturing as well^4,5^. In comparison to many other additive processes, such as additive friction stir deposition^6^ or even fusion-based processes, e.g. selective laser melting, FS does not require a special-purpose machine and can be performed on conventional milling machines^7^ or friction stir welding systems. In terms of the resulting microstructure, the deposited layer material typically shows a significantly refined microstructure enabled by dynamic recrystallization (DRX)^8,9^. As shown in previous studies by the authors on AA5083, multi-layer structures built via MLFS similarly present a homogeneous microstructure^10^ and isotropic mechanical properties^11^.

The available studies on corrosion properties of FS deposited aluminum structures are scarce. However, it is crucial to gain knowledge on the corrosion behavior of FS deposited structures in order to assess the feasibility of this approach for instance for specific repair applications. Yu et al.^12^ performed potentiodynamic polarization and electrochemical impedance spectroscopy for FS of AA6061 deposited over a low carbon steel substrate. The results showed that the refined microstructure of the deposit and randomly distributed intermetallic compounds led to an improved corrosion resistance in comparison to the base material. Farajollahi et al.^13^ investigated the effect of post-weld heat treatment on the polarization curve of FS deposited AA2024 material. The authors reported a decrease of corrosion current after artificially ageing the deposited material. Similar observations were stated by Pirhayati et al.^14^ for FS deposited AA6061. The specific literature available on the corrosion behavior of FS deposited material is not very extensive, but related materials’ processing approaches, which are also based on friction and plastic deformation, like friction stir welding (FSW), show a larger amount of research studies with regard to corrosion^15^. Typically, a FSW joint presents heterogeneous material properties, for instance significant differences in terms of microstructure from stirred zone to base material, resulting in different corrosion behavior^16^. The phenomena in the stirred zone are even more complex when dissimilar materials are welded.

Independent of materials’ processing approach, it has been reported in the literature that the relationship between corrosion and microstructure is complex and strongly dependent on the alloy and the environment. Ralston et al.^17^ investigated the effect of grain sizes achieved via different processing routes on the corrosion behavior of high purity aluminum (99.9%) on the corrosion behavior. For instance, this was shown for equal channel angular pressing (ECAP)^18^, a process based on severe plastic deformation, enabling ultra-fine-grained microstrutures. The ECAP process resulted in redistribution and refinement of the second phase particles within the structure, which is assumed to be one reason for the improved resistance to corrosion as a higher amount of small particles causes a reduction of pitting corrosion for pure Al after ECAP^19^. Another example of a process for materials’ processing involving severe plastic deformation is high pressure torsion (HPT). Ultra fine-grained microstructures of aluminum achieved via HPT were also reported to present an increased corrosion resistance^20,21^. Solid state joining procedures such as FS cause extreme plastic deformation, similar to the example processes of ECAP or HPT. In general, studies for pure Al showed that the corrosion rate tends to decrease for smaller grain sizes; however, the authors^17^ state that more complex alloys lead to additional (chemical) phenomena that affect the corrosion behavior. The type of intermetallic particles in aluminum alloys play a crucial role in passivity breakdown and pit morphology^22^. Different second-phase particles can be found in different aluminum alloys, where the intermetallics’ behavior is mainly affected by the potential difference between the particle and the matrix in solution^23^.

The present study aims for a further understanding of the corrosion behavior of FS deposited material. The FS approach is capable to locally deposit material, where a material of higher corrosion resistance could be chosen in order to protect another alloy. For that purpose, a non-precipitation-hardenable Al-Mg alloy was selected to be deposited onto a high-strength Al-Cu substrate. The MLFS structure generated was used for analyses via potentiodynamic polarization and open circuit potential. The corrosion behavior is directly compared to the consumable stud base material and the results are discussed with perspective to the processing route and microstructural characteristic of the materials. Furthermore, the results are discussed in the view of the findings reported for other processing routes, for instance FSW.

## Materials and methods

### Materials & experimental setup

MLFS layer deposition was performed using a friction welding system (RAS, Henry Loitz Robotik, Germany). The machine has a working space of 1.5 m $\times$ 0.5 m $\times$ 0.5 m (x, y, z) and allows maximum axial forces of 60 kN, maximum torque of 200 Nm and maximum rotational speed of 6000 rpm. All depositions were performed force-controlled and at room temperature. The time between subsequent depositions was long enough to allow the structure to cool down to room temperature. The linear deposition path of 175 mm was programmed via computer numerical control (CNC). The consumable stud material was an AA5083-H112 alloy (20 mm diameter, 125 mm length). The substrate material was an AA2024-T3 alloy (300 mm length, 150 mm width, 8 mm thickness). The chemical composition of the materials is shown in Table 1, respectively. A stack of five layers was built using constant process parameters of 9 kN axial force, 1500 rpm rotational speed and 6 mm/s travel speed, based on the results by Shen et al.^10^.

**Table 1:** Chemical composition of AA5083 and AA2024 (wt%)^24^.

element [wt%]	Al	Cr	Cu	Fe	Mg	Mn	Si	Ti	Zn
AA2024	Balance	$\le$0.10	3.8	$\le$0.50	1.2	0.3	$\le$0.50	$\le$0.15	$\le$0.25
AA5083	Balance	$\le$0.25	$\le$0.10	$\le$0.40	4.5	0.6	–	–	–

**Figure 1:**
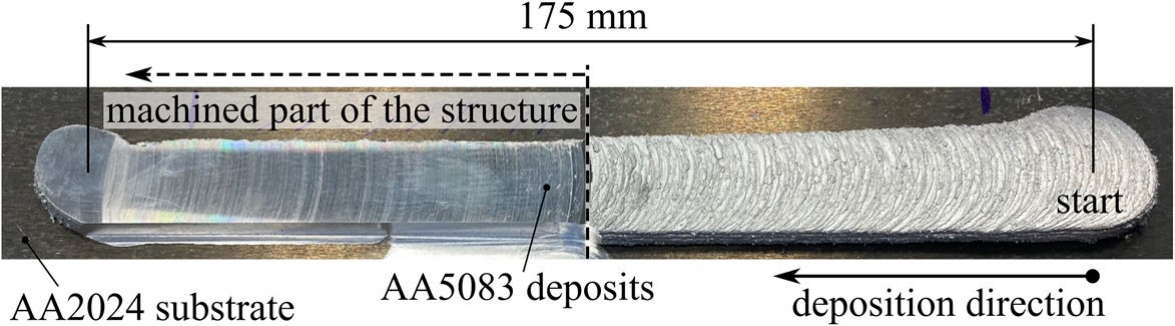
Top view of five-layer multi-layer friction surfacing (MLFS) stack (AA5083) deposited on an AA2024 substrate at constant process parameters of 9 kN axial force, 1500 rpm rotational speed and 6 mm/s travel speed. The pre-programmed deposition path had a length of 175 mm. The second half of the stack was machined in order to remove unbonded parts and the process-characteristic rough surface.

The five-layer MLFS stack deposited at the aforementioned constant process parameters presents a stack height of 6.1 mm and a width of 19.3 mm. The layers show the FS process-characteristic rough surface with some unbonded material on both sides^25^. The stack was partially milled to a height of 5.5 mm and a width of 12.4 mm in order to remove the unbonded material and to achieve a homogeneous surface suitable for the corrosion testing procedures, which are presented in detail in the following. The deposited structure is presented in Fig. 1.

### Methods

#### Sample preparation

**Figure 2:**
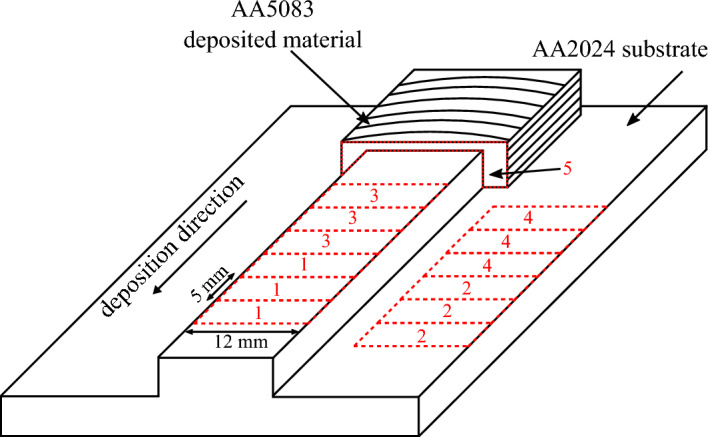
Schematic of multi-layer friction surfacing (MLFS) stack, where one part of the structure was machined. The samples were extracted as follows: #1 (AA5083 FS deposted material) and #2 (AA2024 substrate) for open circuit potential, #3 (AA5083 FS deposted material) and #4 (AA2024 substrate) for cyclic potentiostatic polarization test as well as #5 for microstructure analysis.

For the corrosion testing, the samples (dimension of 12 mm $\times$ 5 mm taken from AA2024 substrate material and AA5083 deposited material) were cold mounted in epoxy resin, where the sides were insulated to avoid crevice corrosion. Fig. 2 shows the schematics of the sample extraction, where corrosion samples were extracted from the machined part of the structure, leaving a gap of 10 mm between unmachined and machined part. For the microstructural investigation, a cross-section was taken from the unmachined part of the structure. Additional samples were taken from consumable stud base material (AA5083-H112) for comparison. The samples for corrosion tests and microstructural investigation were ground with 80 to 1200 grit SiC paper and polished with 4 µm and 1 µm diamond paste. The samples were cleaned in an ultrasonic bath. Afterwards they were cleaned with isopropyl alcohol and dried with nitrogen.

The microstructure analysis in terms of grain size was performed via optical microscopy and electron backscatter diffraction (EBSD) according to standard test methods^26^. The samples were anodized, applying 20 V for 80 seconds in a Barker’s (solution of HBF_4_) etching solution. A scanning electron microscope (SEM), equipped with a energy dispersive X-ray spectroscopy (EDS) and backscattered electrons (BSE), was used to determine the chemical composition and to find possible phases in the the substrate material, the MLFS deposited material and the stud base material, respectively.

#### Cyclic potentiostatic polarization and open circuit potential (OCP)

Electro-chemical tests allow to evaluate the corrosion of the substrate material (AA2024-T3), deposited material (AA5083) and stud material (AA5083-H112). The electro-chemical tests were performed using a 5 mmol/L NaCl aerated solution at room temperature^27,28^. All tests were performed in the submerged sample and using the top surface, which is typically exposed to the environment.

For the cyclic potentiostatic polarization test, a three electrode cell and an Autolab 302N potentiostat were used (On the equipment used (Autolab 302N potentiostat), the cycle polarization test was made after the OCP test, which was measured for 600 s. Due to this programming, the surface of the sample might have changed, which has certain influence on the potentiodynamic behavior for material; however this is constant for all materials tested on this work). The counter electrode (CE) was a Pt gauze and an Ag/AgCl (3 mol/L KCl) was used as reference electrode (RE)^29^. The open circuit potential (OCP) was measured for 600 s before the polarization test. For the substrate and consumable stud base material, the specimens were polarized from -900 mV to 0 mV vs. reference electrode (RE). For the MLFS deposited layer material, the polarization ended at -200 mV and return to -900 mV. After the test, micrographs, obtained using a Leica DM2700 microscope, were analyzed to determine the size of pits in µm^2^ and radius in µm, where the image analysis (a length of 490 pixels accords to 250 µm) was conducted via MATLAB. The same analysis was performed before the polarization test, where no pits were found for all samples investigated. Black particles with a diameter below 2 µm were excluded from analysis to eliminate any potential presence due to dispersoid particles, precipitates, or other phases within the materials used on this work.

The OCP tests of the substrate material, the deposited material and the consumable stud base material were measured for 24 h. The tests were conducted separately for each sample with a fresh solution, where an Ag/AgCl (3 mol/L KCl) was used as the RE^29,30^.

## Results and discussion

### Microscopic analysis

**Figure 3:**
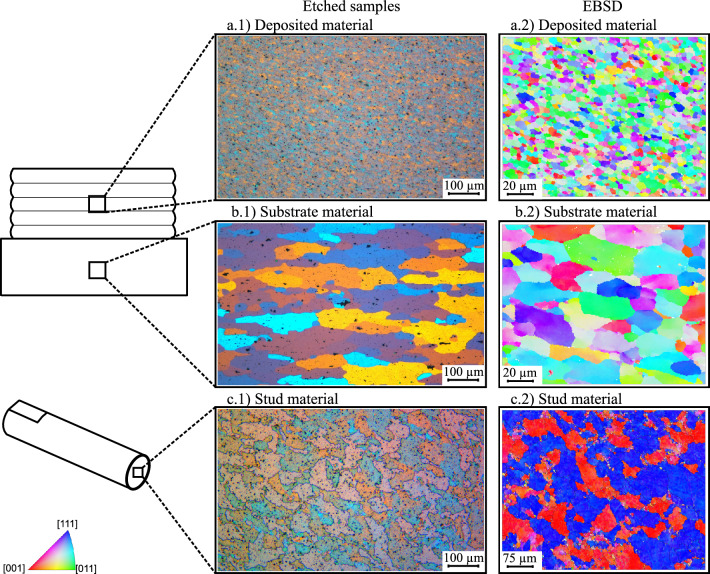
Micrographs, (**a.1,b.1,c.1**) etched (Barker’s solution of HBF_4_) and (**a.2,b.2,c.2**) from electron backscatter diffraction (EBSD), for the microstrutctual analysis of (**a**) AA5083 MLFS deposited material, (**b**) AA2024 substrate material and (**c**) AA5083 stud base material.

Figure 3 shows the etched micrographs of the AA2024 substrate material, AA5083 deposited material and AA5083 consumable stud base material. The substrate material presents a grain size of 63 ±  11 µm in the longitudinal direction and 31 ±  3 µm in the transversal. The stud base material shows an average grain size of 38 ±  7 µm in the longitudinal direction and 32 ±  5 µm in the transversal direction. The deposited material presents almost equiaxed grains with 3.28 ±  1.83 µm. For similar process parameters and materials, Shen et al.^10^ reported average grain size values between 4.0 µm and 4.7 µm, which is in good agreement with Kallien et al.^31^ as well as the results of this study. The grain size within a MLFS stack is relatively homogeneous for every layer^10^. Slight alterations in grain size are linked to the complex material flow during FS layer deposition, leading to local fluctuations in plastic strain and temperature conditions^31^.

**Figure 4:**
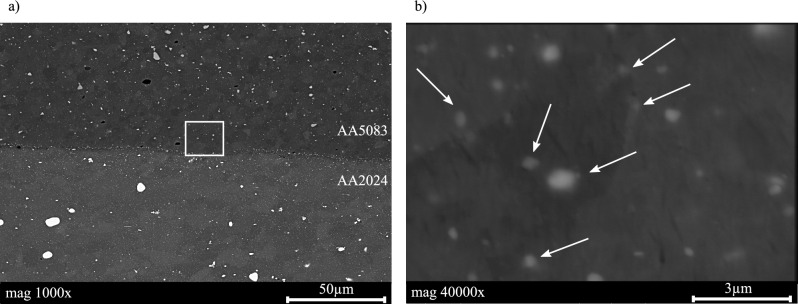
Backscattered electrons (BSE) micrographs of AA5083 deposited onto AA2024 substrate material (**a**). The white box indicates where BSE was performed with higher magnification (**b**). Additionally, arrows point to particles present in the FS deposited material.

Jariyaboon et al.^32^ showed the microstructure of AA2024, which has a composition of precipitation of cooper and the dispersoid particle commonly formed during the homogenisation of the material during the T3 heat treatment. According to Brahami et al.^33^, this dispersoids on AA2024 have a size between 0.1  and 1 µm, where the distribution can be very heterogeneous. It is possible to see these particles in the AA2024 sample of this study, see Fig. 3b, which is in accordance with the literature. EDS was performed in order to locally analyze the chemical composition of the deposited material and substrate. The BSE micrographs confirm the results from EDS, where it was possible to identify particles in the substrate material, presenting 21.3 at.% of copper and 36.3 at.% of aluminium, which could be associated with the Al_2_CuMg S-phase^34,35^. This confirms the precipitation of copper-rich intermetallics in the substrate and is in accordance with literature^35,36^. However, the BSE investigation, Fig. 4a, shows the precipitates on the substrate material and phases present on the MLFS deposited material. Otherwise, the Fig. 4b confirms the presence of $\beta$ phase (Al_3_Mg_2_) on the grain boundaries and all over the grains for the MLFS deposited material.

### Cyclic polarization test

**Figure 5:**
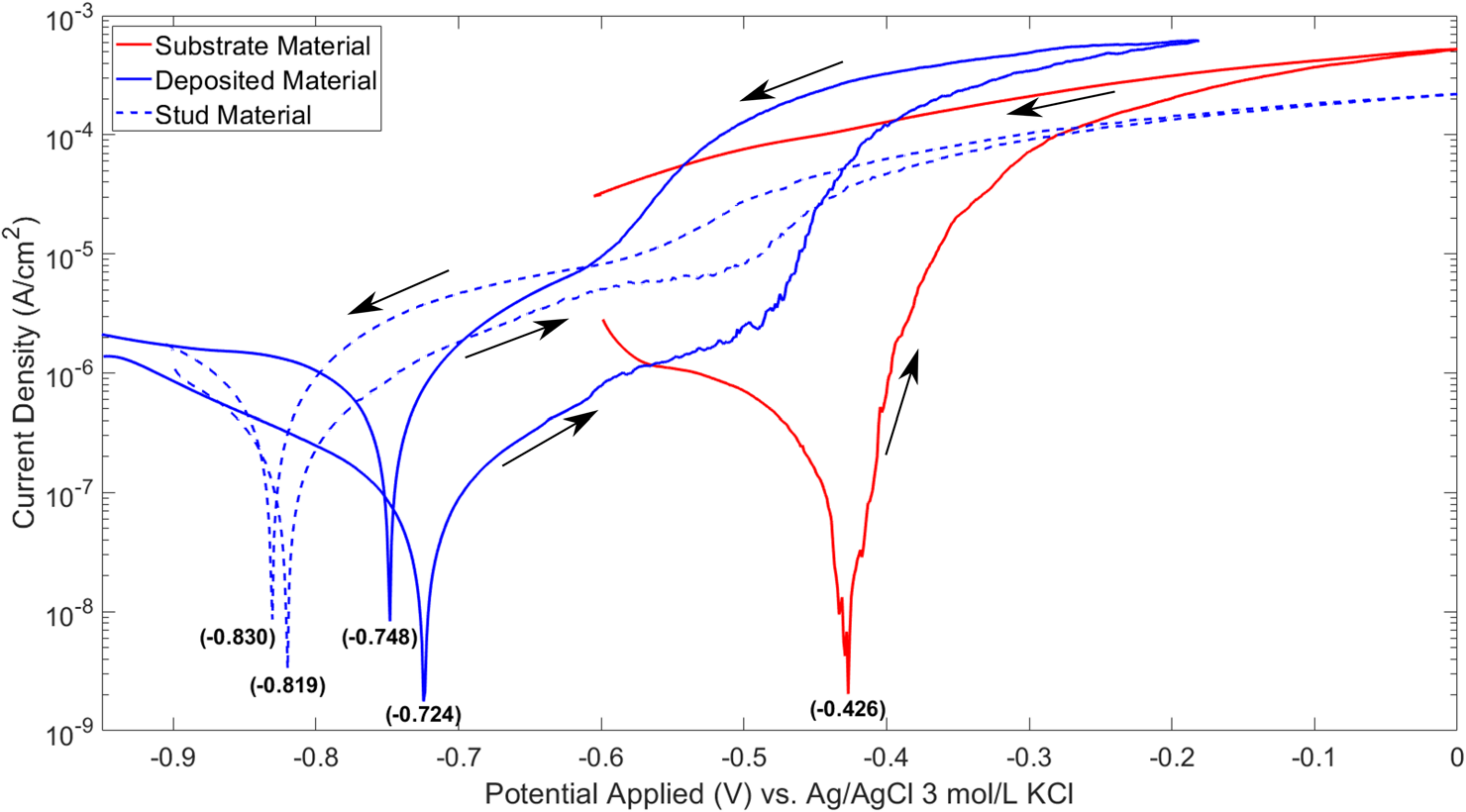
Polarization curves for AA2024 substrate material, AA5083 deposited material and AA5083 stud base material. Ag/AgCl (3 mol/L KCl) was used as reference electrode (RE) and Pt as counter electrode (CE). Current density was measured in A/cm^2^.

Figure 5 shows the behavior of the potentiodynamic polarizations for the materials studied. It is possible to notice a difference of approximately 100 mV in the corrosion potential comparing the stud base material to the deposited material, which showed the same chemical composition. The pitting potential of these two materials is very similar, $-0.527$ V for the stud base material and $-0.492$ V for the deposited material, as shown in Table 2. A higher return hysteresis is observed for the MLFS deposited material in comparison to the stud base material, as well as a repassivation potential value farther from the pitting potential. The differences observed can be related with the difference in grain size; however, both materials, i.e. deposit and respective consumable stud base material, present a passive behavior.

**Table 2:** Potentials for samples from polarization test in solution of 5 mmol/L NaCl.

Samples	Ecor (V)	Epit (V)	Erep (V)
Substrate material	$-0.426$	–	–
Deposited material	$-0.724$	$-0.492$ ± 0.0081	$-0.604$ ± 0.0064
Stud base material	$-0.819$	$-0.527$ ± 0.038	$-0.577$ ± 0.0097

The consumable stud base material and MLFS deposited material did not present a difference in chemical composition for EDS analysis, but the materials are microstructurally distinct^31^, Fig. 3. As mentioned in the introduction, previous works have shown that grain size can play an important role for the material’s corrosion resistance, for instance presented for ECAP^18^ or HPT^20,21^, which are processes causing severe plastic deformation like friction-based solid state materials processing techniques. The FS deposited material has a significantly refined microstructure, Fig. 3b. In this case, the higher density of grain boundaries can lead to the segregation of impurities that act as repassivation obstacles. According to Krishnamurthy et al.^37^, the AA5083 alloy has more susceptibility to intergranular corrosion (IGC) because of precipitation of $\beta$ phase (Al_3_Mg_2_). A fine grained microstructure has higher ratio of grain boundaries and more pronounced corrosion occurs in comparison to the same material with larger grains. The difference between the pitting and corrosion potential indicates the susceptibility to localized corrosion, considering that it marks the free energy required for the nucleation of stable pits. It is observed that the stud base material presents a greater difference between corrosion potential and pitting potential, showing its greater resistance to localized corrosion. A similar observation has been reported by Fahimpour et al.^38^, who showed a poorer corrosion resistance for friction stir welded AA6061 with respect to the base metal. The authors stated that this is an effect caused by the finer grain sizes within the nugget zone.

The AA2024 substrate material, on the other hand, does not show a passive behavior. Therefore, it is inferred that the pitting potential is in the same range as the corrosion potential of the alloy, and stable pits develop from the moment of immersion. Additionally, no repassivation was observed for this material^39^. This behavior is expected for the AA2024 alloy due to the precipitation of intermetallic phases, especially the theta phase^40^.

**Figure 6:**
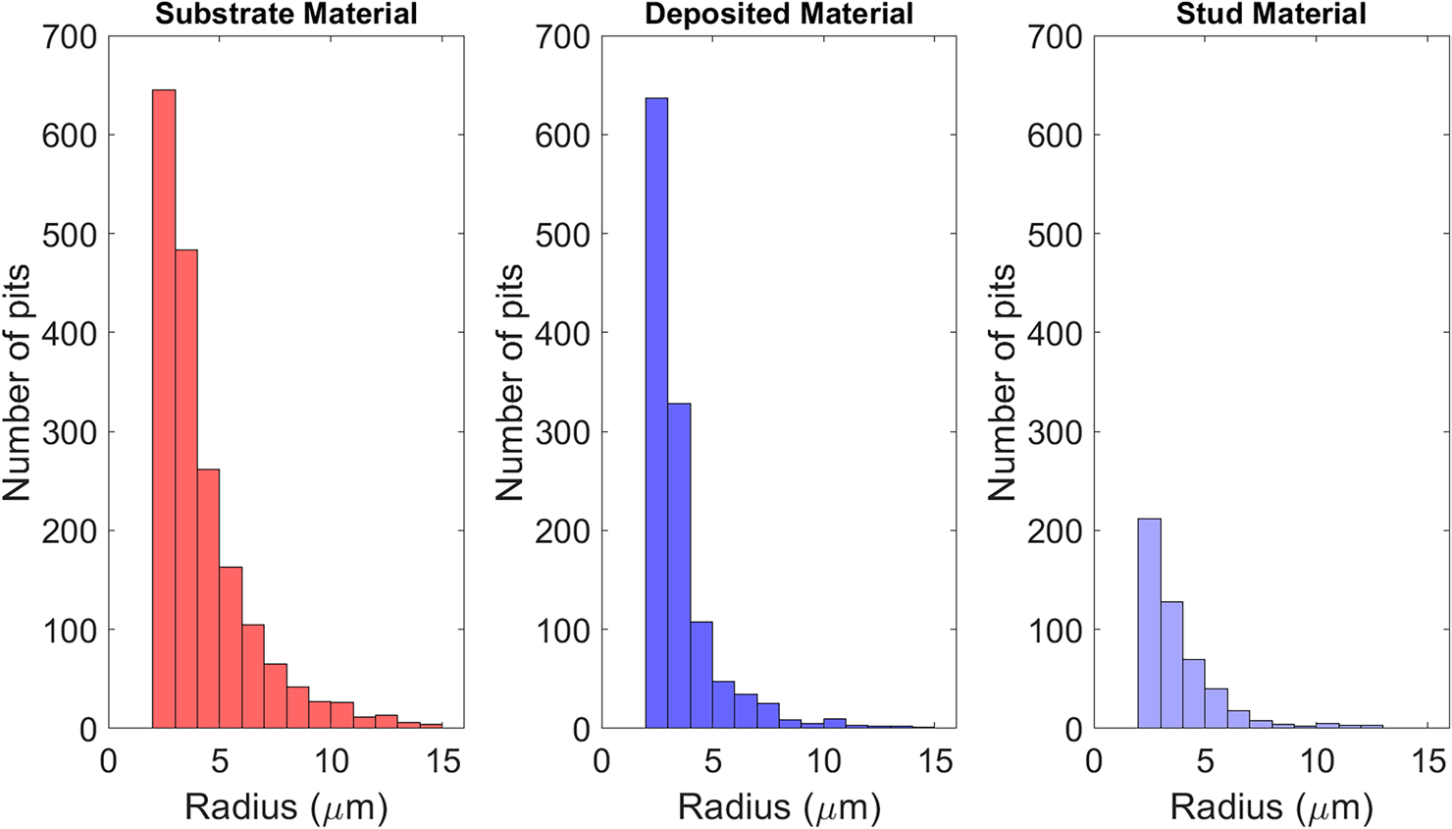
Distribution of pits by number and diameter, for AA2024 substrate material, AA5083 deposited material and AA5083 stud base material after cyclic polarization test.

Figure 6 shows the distribution of pits by number and diameter. It is possible to observe a slight difference between substrate and deposited material. The total number of pits per 250 µm^2^ was 1880 ±  50 on the AA2024 substrate material and 1209 ±  18 on the AA5083 deposited material. This difference might be related to the precipitates present in the substrate material, where corrosion is more propitious to start around copper-rich intermetallics^41,42^.

For the AA5083 stud base material, a much smaller amount of pits was observed (470 ±  22), corroborating with electrochemical tests. Larger pits are more frequently discovered on the AA2024 substrate material as well as the AA5083 deposited material. Nonetheless, there is a noticeable inclination towards more small pits rather than larger ones, showing that for the materials investigated, nucleation predominates over existing pit expansion.

### Open circuit potential

**Figure 7:**
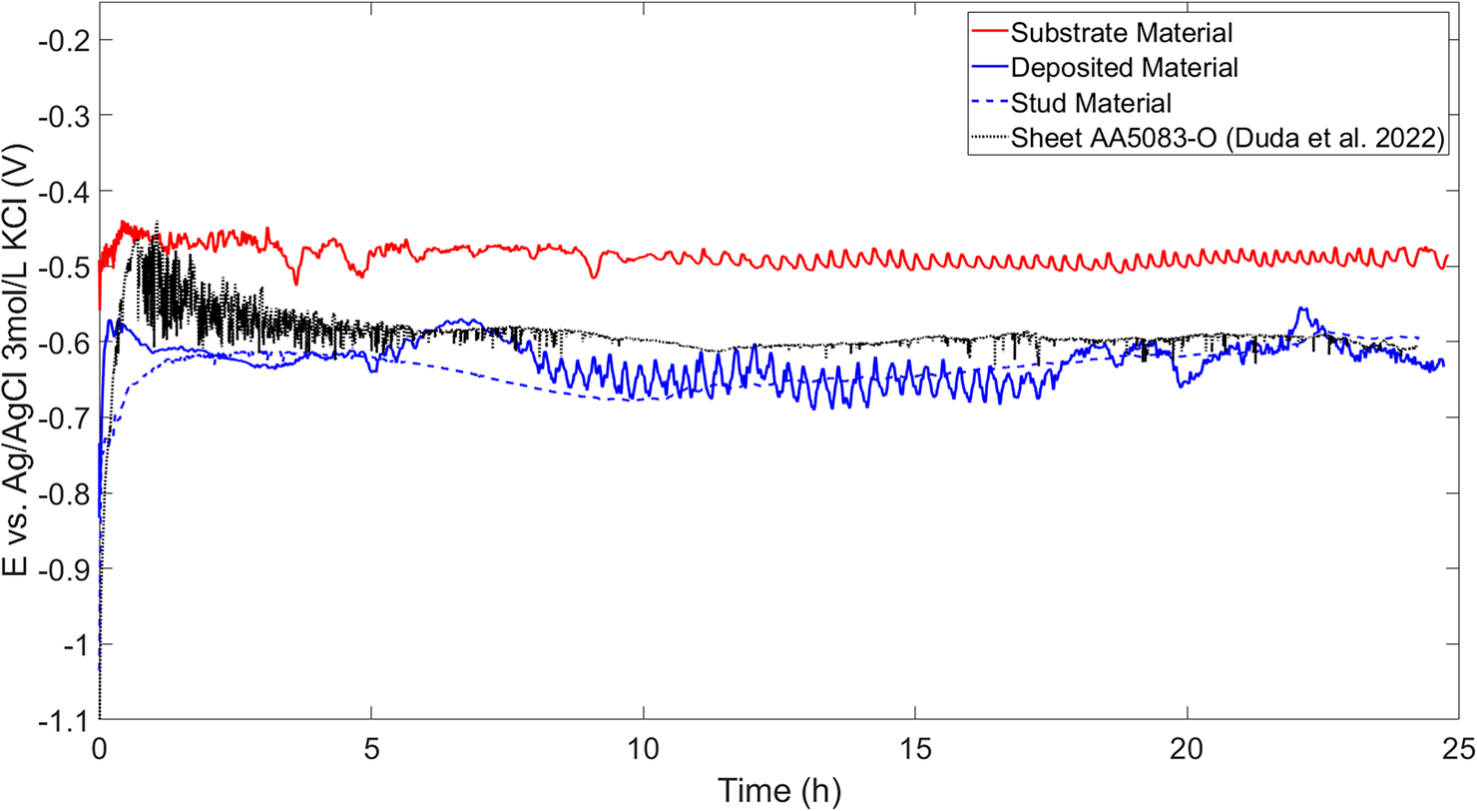
Open circuit potential transient for AA5083 deposited material, AA5083 stud base material, AA5083-O sheet material and AA2024 substrate material in NaCl 5mmol/L aerated. The additional data for AA5083-O sheet material was taken from Duda et al.^45^.

With respect to the 24 h OCP monitoring shown in Fig. 7, it is possible to observe a rapid initial increase for the substrate material, which can be associated with the passive layer^43^. This layer creates a protection for the first 30 min of the test, followed by a slight decrease with oscillating behavior^44^, varying around a steady value throughout the entire immersion. The study performed by Duda et al.^45^ investigated AA5083-O sheet material in same solution, showing a breakage behaviour in the OCP test. At the beginning of the test, the AA5083-O sheet material shows pronounced breakage of passive layer, where, in contrast, the FS deposited AA5083 material is more stable. After approx. 16 h, both materials, the AA5083-O sheet and the FS deposited AA5083 material, have an almost stable value of potential, where FS deposited material shows a slightly lower potential compared to AA5083-O sheet material. This behavior is related to the fact that the AA5083 materials are susceptible to chloride attack^41^. In the case of the consumable stud base material, the oscillating behavior is not observed, corroborating the results of the polarization tests that showed less pits, indicating a greater resistance to localized corrosion for the AA5083 stud base material compared to AA5083-O sheet and AA5083 FS deposited material.

Overall, the different AA5083 materials discussed in this study, i.e. MLFS deposited and machined, AA5083-H112 rod and AA5083-O sheet, underwent significantly different processing routes. Apart from the microstructural characteristic, residual stresses can significantly influence the corrosion behavior of a material as well. For instance, the investigation of Zhang et al.^46^ presented that the machining process can induce large tensile residual stresses, leading to micro-cracking. The study by Takakuwa and Soyama^47^ stated that compressive residual stresses facilitate the passive film formation independent of the surface condition. The enhanced generation and maintenance of the passive film is the reason for improved corrosion resistance^47^. Due to the thermo-mechanical processing, the FS process leads to tensile residual stresses in the deposited layer but induces compressive residual stresses in the remaining sample^48^. However, due to the multi-layer nature of the process, the development and distribution of residual stresses are more complex within the deposited structure. It should be emphasized that no stress relieving heat treatments have been employed. Furthermore, since the residual stresses were not quantified within this study, this aspect needs further investigation in order to be able to judge the influence of residual stresses on the corrosion behavior for MLFS structures, which is beyond the scope of the present study.

## Summary and conclusion

The presented work investigated the corrosion behavior for a dissimilar aluminum multi layer friction surfacing stack. The analyses were performed for the AA2024 substrate material, the MLFS AA5083 deposited material as well as for the respective AA5083 consumable stud base material. The obtained results were discussed with regard to the materials’ microstructure and composition. The main observations can be summarized as follows:

- The AA5083 stud and deposited material differ in terms of grain size, where the deposited material presents a significantly refined microstructure with an average grain size of 3.28 ±  1.83 µm. The fine-grained microstructure presents more grain boundaries, which are more susceptible for corrosion in 5 mmol/L NaCl aerated solution at room temperature.
- The AA5083 deposited material shows a $\beta$ phase (Al_3_Mg_2_) on the grain boundaries and all over the grains, which can result in more pronounced corrosion in comparison to the respective AA5083 stud base material.
- During the cyclic potentiostatic polarization tests, it was shown that the fine-grained MLFS deposited material is more susceptible to nucleation of pits and also showed bigger pits in comparison to the respective consumable stud base material.
- The pit distribution allowed the assessment in terms of material behavior on electrochemical tests, where the AA5083 stud base material was found to be more resistant to corrosion than AA5083 deposited material and AA2024 substrate material.
- OCP showed that the AA5083 stud base material is more stable compared to the AA5083 deposited material, AA5083-O sheet material as well as AA2024 substrate material. For that reason, the consumable stud base material is more noble and resistant to corrosion in the 5 mmol/L NaCl compared to MLFS deposited material.
- The AA2024 substrate material has more and larger pits in comparison with AA5083 when exposed to solution of NaCl. The substrate material has a more severe corrosion compared to deposited material and stud material, which is mainly related to the alloy composition.
- The FS process can be very useful for coating and repair; however, for the materials and corrosion testing environment investigated in this study, the deposited material is less noble than the respective stud base material. However, during OCP monitoring, the AA5083 deposited material showed a similar behavior compared to AA5083-O sheet material.

## Data Availability

The datasets used and/or analyzed during the current study available from the corresponding author on reasonable request.
